# An Experimental Study on the Validity and Reliability of a Smartphone Application to Acquire Temporal Variables during the Single Sit-to-Stand Test with Older Adults

**DOI:** 10.3390/s21062050

**Published:** 2021-03-15

**Authors:** Diogo Luís Marques, Henrique Pereira Neiva, Ivan Miguel Pires, Eftim Zdravevski, Martin Mihajlov, Nuno M. Garcia, Juan Diego Ruiz-Cárdenas, Daniel Almeida Marinho, Mário Cardoso Marques

**Affiliations:** 1 Department of Sport Sciences, University of Beira Interior, 6201-001 Covilhã, Portugal; diogo.marques@ubi.pt (D.L.M.); hpn@ubi.pt (H.P.N.); dmarinho@ubi.pt (D.A.M.); 2 Research Center in Sports Sciences, Health Sciences and Human Development, CIDESD, 6201-001 Covilhã, Portugal; 3 Instituto de Telecomunicações, Universidade da Beira Interior, 6200-001 Covilhã, Portugal; impires@it.ubi.pt (I.M.P.); ngarcia@di.ubi.pt (N.M.G.); 4 Computer Science Department, Polytechnic Institute of Viseu, 3504-510 Viseu, Portugal; 5 Health Sciences Research Unit: Nursing, School of Health, Polytechnic Institute of Viseu, 3504-510 Viseu, Portugal; 6 Faculty of Computer Science and Engineering, University Ss Cyril and Methodius, 1000 Skopje, North Macedonia; eftim.zdravevski@finki.ukim.mk; 7 Laboratory for Open Systems and Networks, Jozef Stefan Institute, 1000 Ljubljana, Slovenia; martin@e5.ijs.si; 8 Physiotherapy Department, Faculty of Health Sciences, Catholic University of Murcia, 30107 Murcia, Spain; jdruiz@ucam.edu

**Keywords:** mobile application, accelerometer sensor, stand-up time, total time, aging

## Abstract

Smartphone sensors have often been proposed as pervasive measurement systems to assess mobility in older adults due to their ease of use and low-cost. This study analyzes a smartphone-based application’s validity and reliability to quantify temporal variables during the single sit-to-stand test with institutionalized older adults. Forty older adults (20 women and 20 men; 78.9 ± 8.6 years) volunteered to participate in this study. All participants performed the single sit-to-stand test. Each sit-to-stand repetition was performed after an acoustic signal was emitted by the smartphone app. All data were acquired simultaneously with a smartphone and a digital video camera. The measured temporal variables were stand-up time and total time. The relative reliability and systematic bias inter-device were assessed using the intraclass correlation coefficient (ICC) and Bland-Altman plots. In contrast, absolute reliability was assessed using the standard error of measurement and coefficient of variation (CV). Inter-device concurrent validity was assessed through correlation analysis. The absolute percent error (APE) and the accuracy were also calculated. The results showed excellent reliability (ICC = 0.92–0.97; CV = 1.85–3.03) and very strong relationships inter-devices for the stand-up time (*r* = 0.94) and the total time (*r* = 0.98). The APE was lower than 6%, and the accuracy was higher than 94%. Based on our data, the findings suggest that the smartphone application is valid and reliable to collect the stand-up time and total time during the single sit-to-stand test with older adults.

## 1. Introduction

As the population’s age increases in industrialized countries [1], older adults’ care is critical for their well-being. Consequently, the evaluation and quantification of daily activities are essential for determining health status changes and, subsequently, detecting early signs of loss of autonomy [2]. Standing up from a sitting position and its counterpart transition, sitting down from a standing position, are the two most common daily motor activities that could be critical indicators for older adults’ functional autonomy [3,4]. The sit-to-stand movement is one of the most challenging activities in terms of mechanics [5]. It requires the optimization of several kinematic tasks, including coordination, balance, mobility, muscular strength, and power output [6]. Within the population of older adults, increased sit-to-stand time is associated with a high risk of fall occurrence [7,8,9], decreased leg muscle power and strength [10,11], slow walking speed [12,13,14], and mobility disability [15]. Usually, the sit-to-stand test’s time combined with the subject’s age and previous medical history (e.g., recovering from an injury or surgery) helps identify the fall risk, assessing functional lower extremity strength, transitional movements, and balance.

Recently, approaches for quantifying mobility emerged that rely on inexpensive sensor technologies [16]. Remarkably, smartphone use has been suggested as a useful tool to objectively monitor and improve patients’ health and fitness [17], which has been verified in research [18] and clinical practice [19]. Smartphone sensor technology has become sufficiently reliable and accurate to substitute specific biomechanics lab equipment and portable devices used in functional mobility research. Some authors showed that a smartphone with a motion sensor could be used as a low-cost integration device to evaluate the patient’s balance and mobility [20]. Other authors demonstrated that the smartphones’ accelerometer could measure kinematic tremor frequencies equivalent to electromyography’s tremor frequency [21]. Wile et al. [22] utilized a smartwatch to differentiate the symptoms in patients with Parkinson’s disease and related tremor diseases by calculating the first four harmonics’ signal power.

Several clinical tests, such as the timed-up and go test and sit-to-stand test, have been developed to evaluate physical performance and mobility-related to every-day tasks [8,23]. The sit-to-stand test is a widely adopted clinical test used to evaluate older adults’ functionality [24]. Initially designed to measure the lower extremities’ functional capacity [25], it has been applied and investigated in different populations to assess the rehabilitation process and functional performance in older people with varied medical conditions [26]. During the sit-to-stand test, researchers and clinicians commonly measure the time spent to perform a fixed number of repetitions or the number of repetitions performed during a specific time [4,14]. Commonly, the evaluators use a chronometer to measure the time during the sit-to-stand test [27,28]. Despite its low cost and ease of use, chronometers present some limitations, mainly associated with human error (e.g., reaction time delay and position judgment) [29,30,31]. Therefore, to overcome the limitations mentioned above, clinicians and researchers may opt for alternative and reliable technologies to measure biomechanical parameters during the sit-to-stand test with aged populations [32,33]. Several authors have analyzed smartphone applications to quantify kinematic variables during the sit-to-stand test using high-speed video recordings [34,35]. Other studies utilized the triaxial accelerometer sensor embedded in the mobile smartphone [30,31,36]. For example, González-Rojas et al. [37] characterized the time measurement of sit-to-stand transitions by transforming the relative acceleration signal, recorded by a triaxial accelerometer. Cerrito et al. [30] validated a smartphone-based app using the accelerometer sensor to quantify the sit-to-stand test movement in older adults. These authors captured vertical ground reaction forces and vertical acceleration simultaneously using two force plates (reference standard) and a mobile smartphone. The total movement duration, peak force, rate of force development, and peak power were measured. Chan et al. [31] also developed a mobile application to calculate the time during the five-repetition sit-to-stand and timed-up and go tests in older women. The mobile application also includes a beep sound to cue the participants to initiate the test, which aims to eliminate potential human errors when using a chronometer, including the reaction time delay.

As frailty is viewed as a transitional state from robustness to functional decline, identifying a pre-frailty state can alleviate or postpone the consequences of this syndrome [38]. Within this context, temporal variables have been used as predictors of frailty in several older adults’ studies. For example, Hausdorff et al. [39] measured the stride time, swing time, stance time, and percentage stance time. Fallers compared with non-fallers revealed higher standard deviations and coefficients of variation across all variables. In the study by Zhou et al. [40], gait is deconstructed into clinically observable spatial-temporal variables to establish a quantitative model to classify fallers and non-fallers.

Considering that the sit-to-stand test is strongly recommended in clinical and research settings to assess functional independence, detect frailty, and sarcopenia in aged populations [15,41], the practicability of using smartphones for assessing the sit-to-stand in older adults can be a logical advancement of an inherent concept. Therefore, as stated above, measuring reliably and accurately temporal variables during the sit-to-stand test with older adults, including the stand-up time and the total time, is determinant to identify those who present functional impairments and designing individual clinical interventions to improve functionality [42,43]. Due to the difficulties of performing the different measurements with older adults, developing an easy-use solution is essential for taking different preventive actions. Therefore, this study aimed to analyze a smartphone application’s validity and reliability to acquire temporal descriptors during the single sit-to-stand test with institutionalized older adults, including the stand-up time and total time. We hypothesized that the smartphone application would be valid and reliable for measuring the temporal variables during the single sit-to-stand test with older adults. This study’s novelty consists of creating a scientifically valid and reliable mobile application for the independent and automatic measurement of temporal variables during the single sit-to-stand test with older adults. To our best knowledge, only one study using a mobile app to quantify the sit-to-stand test with older adults exhibited the data in real-time through graphics [30]. However, analyzing the data through graphics might not be a practical approach in clinical contexts due to its complexity and time-consuming. An essential factor to bear in mind is that clinicians and researchers want to immediately access the results when the test ends to provide feedback in real-time for the participants. The automatic method will also ensure that the data were properly collected. Therefore, with our mobile app, the possibility of automatically recording, processing, and presenting the results in the smartphone screen when the test ends entails a new clinical approach to assess the single sit-to-stand performance with older adults.

## 2. Methods

As part of the research on developing solutions for Ambient Assisted Living (AAL) [44,45,46], the scope of this study consists of using technological equipment that embeds inertial sensors that acquire different data types to measure and identify human movements [47,48,49,50].

### 2.1. Study Design

This study was a cross-sectional design aiming to analyze a smartphone application’s validity and reliability to capture temporal descriptors during the single sit-to-stand test with institutionalized older adults. A digital video camera was used further to validate the correct execution of the sit-to-stand movement, and the results presented in this study only considered the valid ones. The sit-to-stand test is quick and easy to administer and presents practical utility in clinical and research settings to evaluate functional independence in older adults [7,8]. The experimental procedures were carried out over ten weeks. Each session was performed between 10:00 and 11:00 a.m. in the same location (room temperature 22–24 °C). In the first week, we familiarized the participants with the testing procedures. We also measured the body mass (TANITA BC-601, Tokyo, Japan) and height (Portable Stadiometer SECA, Hamburg, Germany). Then, from the second to the tenth week, the participants performed one testing session per week. In each session, we assessed a group of four to five participants in the sit-to-stand test.

### 2.2. Participants

Forty institutionalized older adults (20 men and 20 women) volunteered to participate in this study. Inclusion criteria were age ≥ 65 years old, men and women, capable of stand-up from a chair independently with the arms crossed over the chest, and willingness to participate in the experimental procedures. Exclusion criteria were severe physical and cognitive impairment (i.e., Barthel index score < 60 and mini-mental state examination score < 20), deafness, musculoskeletal injuries in the previous three months, and terminal illness (life expectancy < 6 months). Table 1 presents the characteristics of the participants. All participants gave their informed consent for inclusion before they participated in the study. The study was conducted following the Declaration of Helsinki. The Ethics Committee approved the protocol of the University of Beira Interior (code: CE-UBI-Pj-2019-019).

### 2.3. Sit-to-Stand Test

**Figure 1:**
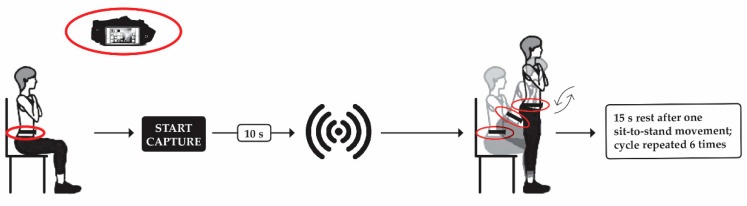
Illustration of the single sit-to-stand test.

For this study, we used the single sit-to-stand test, starting with a 10 min general warm-up consisting of light walking and mobility exercises, as described in Marques et al. [51]. The participants were equipped with a smartphone placed inside a waistband (Sports Waistband Universal Phone Holder), which was in turn attached to the waist. We placed the mobile phone on the waist because the center of gravity is located around the abdomen [52]. The waistband was tightened to avoid slight movements of the mobile phone that would adversely affect data capture. The participants sat on an armless chair (height = 0.49 cm) with the back straight and the arms crossed over the chest. We did not allow the participants to lean back on the chair neither to assume a perched position. All participants were instructed to maintain a 90° hip and knee flexion, which the operator closely monitored during the test. After 10 s of the smartphone application’s activation, an acoustic signal cued the participants to stand up and sit down on the chair while maintaining the arms crossed over the chest, thus performing a single sit-to-stand movement. When they finished the movement, the participant rested on the chair with the arms crossed over the chest for 15 s. The subsequent single sit-to-stand movement was performed after hearing another acoustic signal. After six single sit-to-stand repetitions, the participants had a 3-min rest before repeating the test four more times. This procedure was necessary to ensure that enough and correct data (i.e., having enough rest and without previous body movement before the beep) was collected for post-analysis. Before each sit-to-stand transition, we instructed the participants to perform the repetitions as fast as possible immediately after hearing the acoustic signal. In all trials, a researcher was standing next to the participants to ensure safety during the movement transitions. Figure 1 illustrates the sit-to-stand testing procedure.

### 2.4. Data Acquisition

A smartphone application and a digital video camera acquired the data simultaneously. The latter device was considered the reference criterion [53,54,55]. The smartphone model was the Xiaomi Mi A1. This device embeds a triaxial accelerometer (model Bosch BMI120), which acquires the data at a sampling frequency of 200 Hz. As described before, we placed the smartphone inside a waistband, which was then attached to the participants’ waist. The digital video camera (Canon LEGRIA HF R46, Tokyo, Japan) was positioned perpendicular to the field of view (distance = 3 m) and attached to a stationary tripod (height = 1.2 m). We recorded the participants from the sagittal plane at a sampling frequency of 25 frames per second. Although the sampling frequency between devices was different (200 vs. 25 Hz), this was not considered a significant limitation [53]. In fact, previous studies with older adults comparing the accelerometer data vs. video camera data during postural transitions (e.g., sit-to-stand or gait analysis) used different sampling frequencies between devices [54,55,56]. Therefore, according to the scientific literature, when comparing handheld devices (e.g., mobile phones) vs. machines (e.g., video cameras), it is impossible to achieve synchronization or sampling frequency equality [53].

Regarding using a 25 Hz video camera for analysis, it is essential to note that one frame’s error corresponds to an error of 0.04 s when using this sampling frequency. Table 2 shows that the stand-up time and total time values are around 1.62 and 2.75 s, respectively. These results indicate that the video camera’s error is around 2.5% in the stand-up time (i.e., 0.04/1.68) and 1.5% in the total time (i.e., 0.04/2.75), in the worst-case scenario which means a minor measurement error. Therefore, as stated by Winter [57], except for high-speed running and athletic movements, slower movement analyses (e.g., walking) can be reliably done with minor errors using a 25 Hz video camera. Previous studies with older adults used a sampling frequency of 25 Hz to analyze kinematic data during movement transitions, such as sit-to-stand, stand-to-sit, or sit-to-walk [54,55,56,58,59], which reinforces the validity and reliability of using this frequency for movement analysis.

### 2.5. Data Analysis

#### 2.5.1. Mobile Application

**Figure 2:**
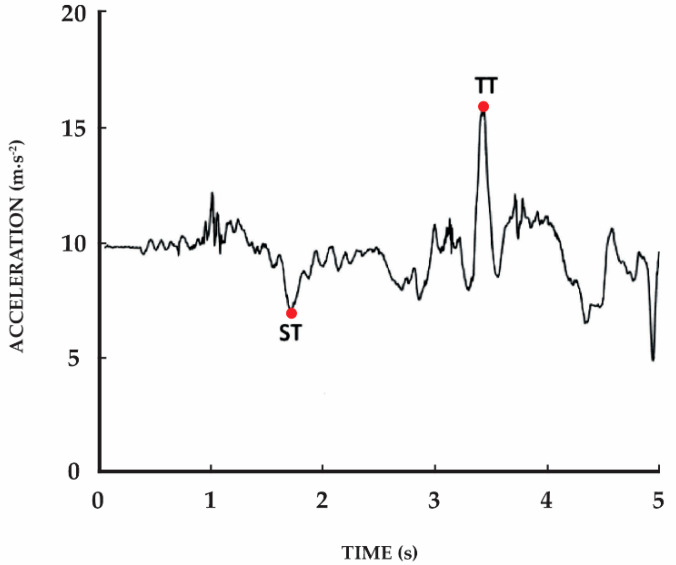
Signal plot of the acceleration during one sit-to-stand repetition (i.e., the complete cycle of stand-up and sit down on the chair); ST: stand-up time; TT: total time.

The accelerometer data were acquired with a mobile application, which automatically pre-processes the raw data and measures the stand-up time and total time (Figure 2). These measures are related to the occurrence of events, such as when standing up starts or ends. The mobile application was developed with Android Studio 4.1., with Java SE 12. It was used for the automatic detection of different events during the sit-to-stand test. It was developed and adjusted, considering the previous study [60], by this study’s research team. The application will be available in the market after validation. Contrary to other studies, and to avoid the mobile device’s incorrect positioning on the waist, we used the Euclidean norm of the accelerometer’s outputs. After, the data was filtered, and the different calculations were applied. The different measurements can be performed locally without an Internet connection. The results are presented as soon as the test ends. The stand-up time starts from the acoustic signal until the minimum (negative) acceleration value is reached before the maximum (positive) acceleration value. The time frame between the acoustic signal and the maximum (positive) acceleration value is defined as the total time (Figure 2). For the measurement of the stand-up time and total time, we researched in the literature how these variables are automatically detected considering the accelerometer data [41,42].

#### 2.5.2. Video-Camera Recordings

The video recording files were transferred to a personal laptop and then analyzed using Adobe Premiere Pro (version 14.4.0, Adobe Systems, San Jose, CA, USA). We analyzed all video files frame by frame. The first frame was considered the start of the acoustic signal. After that, we calculated the stand-up time and total time. The stand-up time was defined as the moment from the acoustic signal until the participant was stand-up with the legs fully extended and an upright torso. The total time was measured from the acoustic signal until the participant returned to the seated position, the moment of contact with the chair, with vertical velocity decreased to zero. We identified that the person was entirely sat by monitoring subsequent frames and ensuring that the last frame corresponded to the moment they were fully seated. We converted the data to seconds by dividing the frame number by 25 frames per second. The repetitions were invalid if participants moved any segment of the body the instant before the acoustic signal or did not complete the sit-to-stand cycle. Therefore, we only selected valid repetitions for further analysis.

### 2.6. Statistical Analysis

The calculation of the interquartile range of the mean difference between devices in each temporal variable enabled the detection of outliers. If data were higher than 1.5 or lower than −1.5 times the Inter-quartile Range (IQR), it was removed [53]. The intraclass correlation coefficient (ICC with 95% confidence intervals [CI]) analyzed the level of agreement or relative reliability inter-device [61]. The ICC model was the two-way random-effects, absolute agreement, single rater/measurement [ICC_(2,1)_] [61]. Cronbach’s alpha analyzed internal consistency. ICC values were interpreted as: <0.50, poor; 0.50–0.75, moderate; 0.75–0.90, good; >0.90, excellent [61]. Bland-Altman plots with 95% limits of agreement (LOA) (mean difference ± 1.96 × standard deviation [SD] of the differences) analyzed the systematic bias/differences between devices [62]. The Kendall Rank Correlation Coefficient (τ) between the absolute differences and the mean of both devices analyzed the degree of heteroscedasticity. If τ > 0.1, the data were considered heteroscedastic and transformed by logarithms to the base 10 (log10) [63]. Linear regressions and Spearman’s Rank Correlation Coefficients (ρ) analyzed the concurrent validity inter-device. The magnitude of correlation was interpreted as: 0.00–0.10, negligible; 0.10–0.39, weak; 0.40–0.69, moderate; 0.70–0.89, strong; 0.90–1.00, very strong [64]. The assumption of homoscedasticity was analyzed by inspecting the standardized residuals’ scatter plots against the standardized predicted values. The absolute reliability was analyzed by estimating the standard error of measurement (SEM = SD of the difference between the smartphone application and video camera scores divided by the √2), the coefficient of variation (CV = (SEM/mean of both devices) × 100), and minimal detectable change (MDC = √2 × SEM × 1.96) [65]. CV values < 5% were considered acceptable [66]. The absolute percent error of the measurements (APE = ((|smartphone application − video camera)/video camera|) × 100) [67], and the accuracy ((video camera − (|video camera − smartphone application|)/video camera) × 100) were also calculated. An APE < 10% was considered acceptable [67]. We conducted a sample size calculation based on an expected reliability level of 0.90 and a minimum acceptable reliability level of 0.80. With an alpha value of 0.05 and 6 repetitions per participant, a minimum sample size of 32 was required to obtain a power of 80% [68]. The significance level was set at *p* < 0.05. All data were analyzed using Microsoft Office Excel 2016 and SPSS version 27 (SPSS Inc., Chicago, IL, USA). Figures were designed using the GraphPad Prism version 7.0 (GraphPad Software Inc., San Diego, CA, USA).

## 3. Results

**Figure 3:**
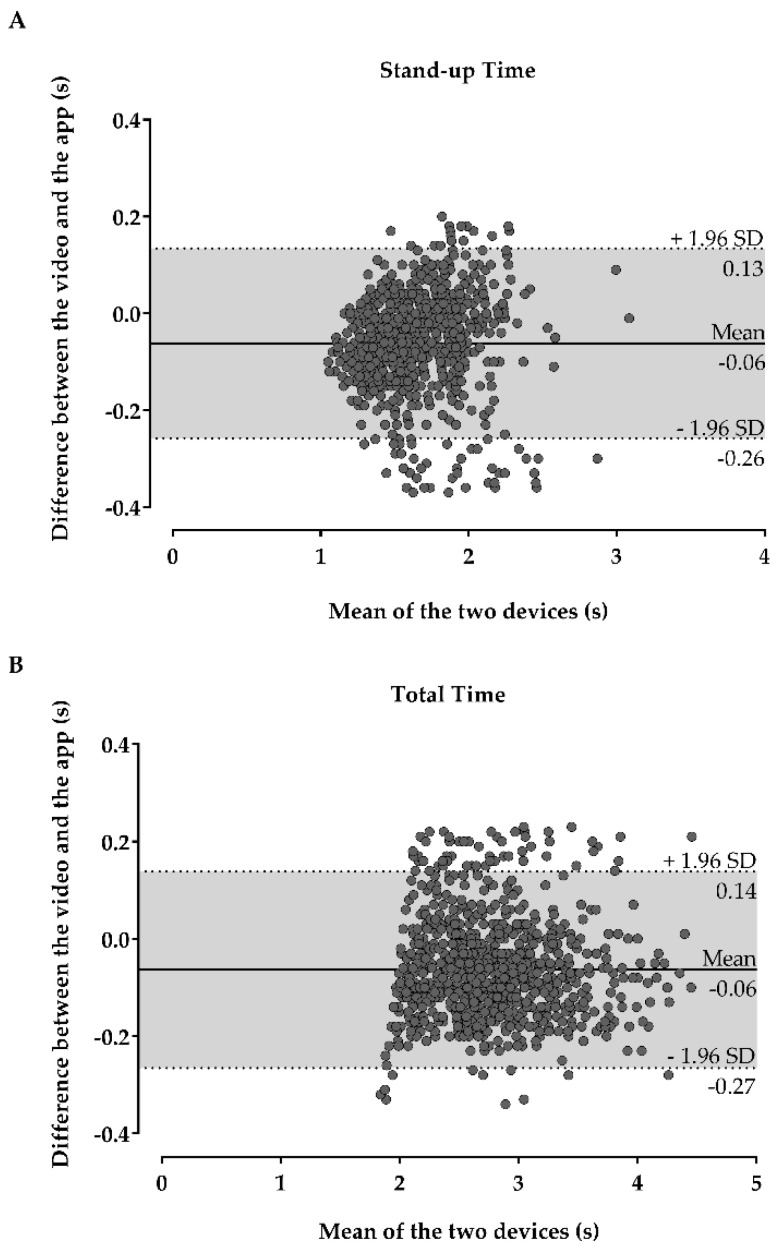
Bland-Altman plots with 95% limits of agreement (mean difference ± 1.96 × standard deviation [SD] of the differences) between the mobile application and video-camera for the stand-up time (**A**) and total time (**B**); the solid lines in the middle of the plots represent the mean difference/bias, while the upper and lower dotted lines represent the upper and lower LOA.

**Figure 4:**
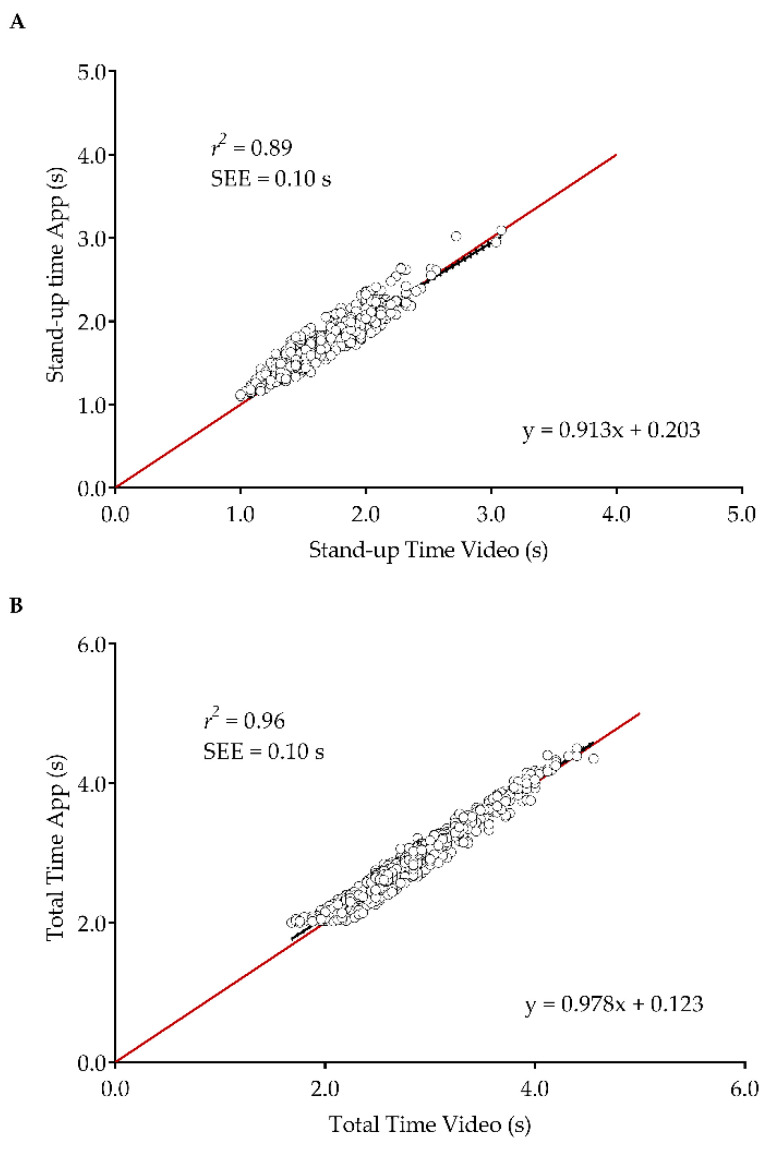
Linear regression between the mobile application and video-camera for the stand-up time (**A**) and total time (**B**); *r*^2^: coefficient of determination; the black lines indicate the regression line, while the red lines indicate the line of 45°; dotted lines indicate 95% confidence intervals.

Table 2 presents the relative reliability and relationship between devices. The results obtained through manual based on the video camera recordings correspond to traditional methods when an operator observes the patient performing the test and times his/her movements with a chronometer. It is expected that the careful analysis frame-by-frame to measure the time of the test is more accurate than an operator measuring the time with a chronometer. Therefore, the results provided in Table 2 and Table 3 and Figure 3 and Figure 4 comparing the video camera results and the smartphone-based approach correspond to a comparison between a traditional and a smartphone-based approach as well. The stand-up time and total time showed excellent relative reliability and very strong significant relationships (*p* < 0.001) inter-devices.

Figure 3 shows the Bland-Altman plots of agreement between the mobile application and video-camera for the stand-up time (A) and total time (B).

Figure 4 shows the linear regression between the mobile application and the video-camera for all variables. The line of 45° indicates the amount of difference inter-devices in the measurement of the variables. Both the stand-up time and total time fall nearby the line of 45°. The resulting linear regression equation is provided for both variables.

Table 3 presents the absolute reliability and accuracy between devices. The stand-up time and total time showed CV values lower than 4%, revealing excellent absolute reliability. In both variables, the APE values were lower than 6%, and the accuracy was higher than 94%, thus revealing a high accuracy level.

## 4. Discussion

In this study, we analyzed the validity of a mobile smartphone application to quantify the stand-up time and total time during the single sit-to-stand test with institutionalized older adults. The results revealed excellent reliability, high accuracy, and very strong relationships between devices in both temporal variables. These results agree with our central hypothesis, meaning that the mobile application is valid and reliable for measuring temporal variables during the single sit-to-stand test with institutionalized older adults.

Regarding stand-up time, only two studies reported acquiring this temporal variable with a mobile application during the sit-to-stand test with older adults. In a study with stroke patients [36] of both sexes (67.50 ± 13.18 years), the authors could observe a mean stand-up time of 1.95 s (SD = 0.08), which is 16% longer than the value observed in our study (1.68 ± 0.29 s). With our study focusing on older adults without any medical conditions that would affect mobility during the sit-to-stand test, these differences are expected. In the second study [30], which included community-dwelling older adults (73.5 ± 10.4 years), the reported mean stand-up time of 1.66 s (SD = 0.42) is close to our results. Possible reasons for these similarities might be associated with using a triaxial accelerometer embedded in the smartphone, a similar system to develop the mobile application, the participants’ age, and the maximal intended speed during the sit-to-stand transfer. Other studies that captured the stand-up time through a mobile application [35] reported significantly lower times (0.47 ± 0.09 s). However, the results are incomparable as the participant sample pool included adults of a significantly wider age range (21–87 years), and the stand-up time in this study was defined as the rising phase of the sit-to-stand movement without taking into account the preparatory phase, i.e., when the trunk is shifted forward prior to seat-off. Additionally, the mobile application developed in that study quantified the sit-to-stand test based on high-speed video recordings and not through the triaxial accelerometer incorporated in the smartphone. Having only three studies related to the sit-to-stand test and its analysis using mobile devices suggests that this is an active area of research. Furthermore, the inconsistent results reported by different studies due to the reasons mentioned above justify the work performed in our research, which is in a more controlled and homogeneous age group of older adults, lacking in other studies.

Studies that used body-fixed sensors instead of a mobile device to record the stand-up time with older adults observed that the time ranged between 1.81 to 2.17 s [69,70,71,72]. Furthermore, when the participants were instructed to perform the movement as fast as possible, the time decreased to 1.74 s (SD = 0.33) [69] and 1.7 s (SD = 0.80) [42]. These results are relevantly like the stand-up time values presented in our study, mainly when the sit-to-stand transfer is performed at the maximal intended velocity. Considering the findings above, the accelerometer data acquired with a hybrid sensor or a mobile application seems to present similar results in the sit-to-stand test with older adults.

Regarding the total time (i.e., the complete measurement of the stand-up/sit-down cycle), to our best knowledge, only one study measured this variable with a mobile application [36]. Merchán-Baeza et al. [36], in a study with stroke patients, reported a total time of 4.09 ± 0.07 s. This time is 45% higher than the total time presented in our study (2.81 ± 0.50 s). However, this result is expected as none of our participants had suffered from a stroke. Hence, the observation of a faster time for sit-to-stand transitions in older adults without mobility disability when compared to stroke patients can be anticipated.

Our mobile application demonstrated high accuracy levels and minor errors to capture temporal variables during the single sit-to-stand test with older adults, reinforcing its validity and reliability. Although several related studies mentioned accurate mobile apps to measure temporal variables during the sit-to-stand test with older adults [31,35], none reported the accuracy values like ours, which does not allow valid comparisons with our results.

We want to note that the paper discusses a mobile app designed primarily for use in clinical settings or in controlled settings when the patient has help from caregivers, medical personnel, or family members to set up the application and place the mobile device properly. If used in an uncontrolled environment, then the test results can be invalid. However, even in such limiting circumstances, not having to visit a medical center to perform the test is quite valuable for older adults with mobility and dexterity problems.

Future studies should consider the following limitations and perform new analyses in the sit-to-stand test with aged populations to strengthen this field’s knowledge. Firstly, the sit-to-stand test analysis can be strengthened by capturing other biomechanical variables. For example, developing an algorithm to calculate the velocity, force, and power generated during the sit-to-stand transitions will provide insightful information for researchers and clinicians. Secondly, determining the mobile application’s intra-device reliability by repeating the experiment over different trials will help analyze the results’ consistency. Thirdly, analyzing the mobile app’s validity and reliability considering smartphones with different sampling frequencies will help understand if its use can be generalized among several devices. Finally, applying other field-based tests such as upper and lower body strength tests, the timed-up and go, or walking speed tests will enable researchers to analyze their relationship with the temporal variables collected during the sit-to-stand test.

Another promising avenue for research is to also include capabilities in the mobile application to identify whether the test was performed correctly, such as due to incorrect body positioning or performing incomplete movements. For the current research, we assume that the participants were trained to perform the test when asked to install the mobile application. Additionally, the mobile app will also include informative videos and tutorials about the test’s procedures.

## 5. Conclusions

For older adults, sit-to-stand tasks are an essential facet of independence and well-being. Therefore, improved quantification of the sit-to-stand test is warranted. It can provide important information that can help improve the quality of life of older adults. The smartphone application presented in this study is suitable for valid and reliable measurements of temporal variables during the single sit-to-stand test with institutionalized older adults, specifically, stand-up time and total time. Researchers and clinicians commonly use these variables for different purposes, such as identifying frailty and analyzing the effects of different training interventions on these variables (i.e., do they improve the time to stand-up from the chair after a training program?). Therefore, having a valid and reliable instrument like our mobile application to measure these variables is clinically essential for capturing accurate data. The smartphone application can also be used in contexts where budget, space, time, and equipment are limited. It is also essential to note that, as the test ends, the results are presented on the smartphone screen in real-time, meaning that the evaluators can immediately access the data and provide reliable feedback regarding the test’s performance. As a result, there is no need to use other materials more sensitive to human error like chronometers to capture the data. Finally, the data is also stored in the mobile phone and cloud, enabling follow-up analysis.

In the future, these tests should be evaluated by multidisciplinary teams comprised of coaches, physiotherapists, physicians, nurses, and technicians to identify potential issues that might have been neglected during this study. A pilot test in a broader population performed for a prolonged period should be conducted to evaluate the long-term effects of exercise or rehabilitation on older adults’ sit-to-stand performance. As a result, it would help determine whether the current practice should be modified or updated and under which conditions.

## Figures and Tables

**Table 1:** Participants’ characteristics.

	*n*	Age (Years)	Body Mass (kg)	Height (m)	BMI (kg/m^2^)
Women	20	81.9 ± 8.1	65.4 ± 11.6	1.49 ± 0.1	29.4 ± 5.4
Men	20	76.0 ± 8.2	78.0 ± 15.5	1.66 ± 0.1	28.4 ± 4.7
Total	40	78.9 ± 8.6	71.7 ± 15.0	1.57 ± 0.1	28.9 ± 5.0

Data are mean ± standard deviation.

**Table 2:** Relative reliability and relationship inter-devices.

Variable	App (s) (Mean ± SD)	Video (s) (Mean ± SD)	Cronbach’s α	ICC (95% CI)	Correlation (ρ) (95% CI)
Stand-up Time (*n* = 842)	1.68 ± 0.29	1.62 ± 0.30	0.97	0.92 (0.82–0.96)	0.94 (0.94–0.95) ***
Total Time (*n* = 892)	2.81 ± 0.50	2.75 ± 0.50	0.99	0.97 (0.93–0.98)	0.98 (0.97–0.98) ***

CI: confidence interval; ICC: intra-class correlation coefficient; ρ: Spearman’s rank correlation coefficient; *** *p*-value < 0.001.

**Table 3:** Absolute reliability and accuracy inter-devices.

	Stand-Up Time (*n* = 842)	Total Time (*n* = 892)
SEM (s)	0.05	0.05
CV (%)	3.03	1.85
MDC (s)	0.14	0.14
APE (%)	5.79	3.90
Accuracy (%)	94.21	96.10

APE: absolute percent error calculated as: ((|smartphone application − video camera)/video camera|) × 100); Accuracy calculated as: ((video camera − (|video camera − smartphone application|)/video camera) × 100); CV: coefficient of variation; MDC: minimal detectable change; SEM: standard error of measurement.

## Data Availability

Data is available in Mendeley data at http://dx.doi.org/10.17632/335rmgrfw2.4 io (accessed on 29 September 2020).
